# Annotations

**Published:** 1890-07-12

**Authors:** 


### Annotations.

**International Hygiene.** At the Mansion House last week, a meeting was held under the presidency of the Lord Mayor to awaken interest in the forthcoming International Congress of Hygiene. The meeting was thoroughly representative, and included men of the highest scientific eminence, both of the medical and other professions. Professor Humphrey, of Cambridge, in seconding the first resolution, said he did so "because he considered that England ought not to be backward in matters that so largely affected the happiness of humanity. *Salus populi suprema lex*: it was so for many reasons. Health was the avenue to happiness, and a vigorous and healthy body was a requisite for a vigorous and healthy mind, and what was of not less importance, a healthy morale. The country which was first in sanitary conditions was first in commercial success." The people of this country have long been aware of the value of sound health and of the conditions necessary to secure it. Sanitarians, led by the late venerable Sir Edwin Chadwick, have played the part of health apostles for many years. It is more than satisfactory that all medical men who can pretend to any intelligence are joining the army of the sanitarians and declaring that it is as necessary to teach the public the methods of individual and personal sanitation as to teach them the arts of drainage and the general sanitation of houses and streets. If we once recognize that sanitary learning and science are under obligations to those who are ignorant, to teach them what they should do, we cannot stop short at the purifying of streets and the sweetening of houses. The more important part of the same business is the instruction of individuals in personal hygiene. If we take up sanitation at all on behalf of the public we are bound to carry our theories to their logical issue; and that logical issue is the giving to the people in such a form as they can understand it, all the knowledge needful for their sound and perfect health. Foolish and inexperienced persons may say that if the people are so well instructed in their own management they will have no need of doctors. Our contention is and always has been that the demand for doctors will increase in the exact proportion that general knowledge of physiology and medical science increases. Religion spreads with ten times greater rapidity among the instructed than among the ignorant. So will medicine be in infinitely greater demand when men know the risks they run by permitting themselves to fall into ill-health, and the dangers they incur when they attempt to tamper, without special training, with the powerful and often poisonous substances employed by the modern scientist for their healing. Not only so, but medical science will be far more effective in operation when the intelligence of the patient and of his household is enlisted on the physician's side. By all means, in every department of human life, "Let knowledge circle with the suns."

**A Terrible Procession.** On the subject of Infant Assurance Mr. Benjamin Waugh, whose experience and judgment are to be thoroughly relied upon, has taken up the position that the only reform which will deal thoroughly and efficiently with the question is to abolish such insurance altogether. Many peoples' minds have been gradually inclining in that direction, and those who are still wavering may find some help to decision in the following facts related in a morning contemporary of July 5th. "The Chief Constable of Rotherham gave evidence yesterday before the Committee of the House of Lords on this question, presided over by the Bishop of Peterborough. A few days ago the witness prosecuted a dissolute man and his wife for neglect of their six children. These were found out by one of his detectives and taken to the workhouse. They were in a most emaciated condition, almost moribund, and one fifteen months old, which was not expected to survive, was photographed. The photograph was shown to the Committee. On searching the house where the children had been kept by their parents, insurance policies were found, proving that if the children died within a given period the parents would receive in all £28 17s.; though they would have paid only 7s. . . . . Competition among insurance agents was so keen that he had heard that midwives were induced to inform them whenever a child was born . . . . The Medical Officer of Health for Leek gave statistics showing that when the local burial society discontinued the practice of insuring children under twelve months old infant mortality fell off, and that when the practice was taken up by insurance companies the mortality rose." There never yet was a practice so bad but that something might be said for it, and a very plausible story may be made out by those insurance societies who do the business of child insurance. But we do not hesitate to say that it will be better to bury every dead child, whose parents are too poor to pay for its funeral, out of the rates, than that our country should endure the intolerable scandal of wide-spread child murder. The picture of six helpless infants in one wretched house, being slowly starved to death, in order that the brutal and besotted parents might at one stroke secure freedom from all parental responsibility, and the means of procuring a prolonged orgie in drink, cannot but appeal to the imagination of the very dullest. When it is remembered that this kind of thing is going on in every large and small town of the United Kingdom, and that our population numbers not less than 36,000,000, there is painted before the mind's eye a scene which is perfectly appalling and overwhelming; a picture of young children less than a year old, numbered, not by thousands, but by tens of thousands, dying by inches in every stage of slow starvation. This is what is taking place to-day in every part of our country; and if the waving of a magician's wand could bring all these helpless little ones into slow procession to Hyde Park, what a heart-breaking demonstration would be there? The greed of people to be rich is infamous and horrible, and leads to consequences which, if they were not manifest, would be absolutely incredible. We must do something, not merely to mitigate, but to put an end to a national disgrace which cannot be endured. If the Lords' Committee cannot do something more thorough than they have done for the sweaters' victims, the House of Lords will do well to appoint no more committees, and to raise no more vain hopes for the mere purpose of deluding them. We live in a period of Christian civilisation; and if Christian civilisation cannot put an end to the wholesale murder of helpless infants, what is it worth?

**State Logic.** The Housing of the Working Classes Acts Amendment Bill promises to be a satisfactory measure. Members of Parliament as well as others are gradually emancipating themselves from the iron bondage of abstract political economy. It is curious and instructive to note how in religion, in philosophy, in politics, and in social questions men have first generalised from experience, and then elevated their generalisations into dogmas which must neither be disputed in argument nor deviated from in practice. Now, general principles are excellent things for general guidance. They are like highways through a country, and greatly facilitate all kinds of business. But a distinction must be made among principles, and it is a dangerous thing to mix up the expedient and the accidental with the absolute and the essential. Every man should be just, and every man should provide for his own wants. Of these two principles the one is quite as sound as the other, but the one is by no means so capable of universal application as the other. Every man can always be just, if he will; but every man cannot always provide for his own wants, however resolute and honest his will may be. A complicated civilization is a civilization surrounded by limitations and barriers, strong as iron and grim as death. Hence it is that we have to call in an apparent and peculiar principle, that of mutual regard and mutual help. This principle, though unknown to political economy, is not really peculiar, but is in universal operation among men. Acting upon it we provide hospitals for the sick. And the very same law of sympathy which makes us provide hospitals for the sick impels us to provide healthy homes for the working classes whenever it happens that they are not able to provide such homes for themselves. Some well-intentioned people think that the State should not recognise the necessities of one class more than those of another, and this is a theory which can be made to look both logical and plausible. But the State, be it remembered, is nothing but the voice and hand of the community, and the State would be as much justified in leaving the pauper to starve in the gutter and the sick to die in the street as it would be in leaving the working classes to slowly ruin their health in pestiferous houses when they have not the power to provide homes for themselves of any other kind.

